# Global Registries in Congenital Hyperinsulinism

**DOI:** 10.3389/fendo.2022.876903

**Published:** 2022-06-02

**Authors:** Tai L. S. Pasquini, Mahlet Mesfin, Jennifer Schmitt, Julie Raskin

**Affiliations:** Congenital Hyperinsulinism International, Glen Ridge, NJ, United States

**Keywords:** rare disease, registry, congenital hyperinsulinism, hypoglycemia, patient-reported outcomes

## Abstract

Congenital hyperinsulinism (HI) is the most frequent cause of severe, persistent hypoglycemia in newborn babies and children. There are many areas of need for HI research. Some of the most critical needs include describing the natural history of the disease, research leading to new and better treatments, and identifying and managing hypoglycemia before it is prolonged and causes brain damage or death. Patient-reported data provides a basis for understanding the day-to-day experience of living with HI. Commonly identified goals of registries include performing natural history studies, establishing a network for future product and treatment studies, and supporting patients and families to offer more successful and coordinated care. Congenital Hyperinsulinism International (CHI) created the HI Global Registry (HIGR) in October 2018 as the first global patient-powered hyperinsulinism registry. The registry consists of thirteen surveys made up of questions about the patient’s experience with HI over their lifetime. An international team of HI experts, including family members of children with HI, advocates, clinicians, and researchers, developed the survey questions. HIGR is managed by CHI and advised by internationally recognized HI patient advocates and experts. This paper aims to characterize HI through the experience of individuals who live with it. This paper includes descriptive statistics on the birthing experience, hospitalizations, medication management, feeding challenges, experiences with glucose monitoring devices, and the overall disease burden to provide insights into the current data in HIGR and demonstrate the potential areas of future research. As of January 2022, 344 respondents from 37 countries consented to participate in HIGR. Parents or guardians of individuals living with HI represented 83.9% of the respondents, 15.3% were individuals living with HI. Data from HIGR has already provided insight into access challenges, patients’ and caregivers’ quality of life, and to inform clinical trial research programs. Data is also available to researchers seeking to study the pathophysiology of HI retrospectively or to design prospective trials related to improving HI patient outcomes. Understanding the natural history of the disease can also guide standards of care. The data generated through HIGR provides an opportunity to improve the lives of all those affected by HI.

## Introduction

Congenital hyperinsulinism (HI) is the most frequent cause of severe, persistent hypoglycemia in newborn babies and children (1–3). In most countries, HI occurs in approximately 1/25,000 to 1/50,000 births (4). About 60% of babies with HI develop hypoglycemia during the first month of life. When HI is not recognized and diagnosed or if treatment is ineffective in preventing hypoglycemia, brain damage or death can occur (1– 4). Although some aspects of HI are well established, many questions remain unanswered. In rare diseases, registries provide an opportunity to investigate the many unanswered research questions and help identify individuals who may be interested in future study opportunities (5–7). Congenital Hyperinsulinism International (CHI) created the HI Global Registry (HIGR) in October 2018 to collect patient-powered HI insights. For HI, HIGR provides the foundation for new research areas into the natural history and burden of the disease.

## Background

In its simplest form, a registry is a tool to collect data. Commonly identified goals include formalizing a contact list, performing natural history studies, establishing a network for future product and treatment studies, and supporting patients to offer more successful and coordinated care (5–8).

Patient-reported outcomes refer to data elements reported by patients or their representatives (6, 9, 10). Regulators have signaled an increased acceptance of the use of real-world data and patient-reported outcomes as part of clinical trial designs and regulatory decision-making (11, 12). Members of the rare disease community focus on increasing trust, especially among regulators, in the validity and use of patient-reported data (11). The use and acceptance of registries for generating data are growing, and for some diseases, patient registries may provide the most current, relevant, and in-depth research for the condition.

Patients and caregivers are uniquely positioned to provide information related to their symptoms, functional status, quality of life, and the overall burden of disease (5). Skepticism related to patient-generated data quality has subsided in recent years as rigor has increased through implementing best practices in governance, design, and data quality (13–15). Very little is known about most rare diseases, and registries can be a critical tool in providing additional insight into the medical and day-to-day experience of living with a rare disease.

## Knowledge Gaps in HI

In 2020, CHI launched the Collaborative Research Network (CRN) to establish a global network of HI experts to create a patient-focused prioritized research agenda and to formalize an infrastructure for collaboration to conduct HI research. The goals of the CRN include improving the time and accuracy of a diagnosis, driving new evidence-based treatments and cures, standardizing clinical guidelines, and facilitating improved access to treatments. The CHI CRN Council includes 56 academic and biotech researchers, clinicians, and patient leaders from 19 countries.

Through the process of building the prioritized research agenda, some of the significant gaps identified included understanding the effectiveness of current treatment options, data to support the evolving standards of care for HI patients, determining global prevalence, more information on associated comorbidities of HI, and to better understand the role of timely diagnosis on developmental outcomes. This international consortium identified HIGR as an important tool for generating new insights into the natural history of HI, medication side effects, diet and feeding habits, glucose levels, the patient experience and quality of life, and the overall burden of disease.

## Methods

The HI Global Registry (HIGR) was launched in October 2018 by Congenital Hyperinsulinism International (CHI), a nonprofit patient advocacy organization dedicated to research, awareness, and support. CHI is responsible for the day-to-day data collection, recruitment, strategic direction, and stewardship of HIGR, the only global patient-powered HI registry. The registry utilizes the IAMRARE™ online platform hosted by the National Organization for Rare Disorders and developed with input from FDA, NIH, patients, organizations, and experts in the field.

The registry is guided by a research protocol approved by an institutional review board (IRB) and advised by internationally recognized HI patient advocates and experts, known as the HI Global Registry Steering Committee. Privacy management and data security principles are followed to protect the personal data of all participants. The goal of HIGR is to advance the global understanding of HI and drive research toward better treatments and, ultimately a cure.

The primary objectives of HIGR are:

- To provide a convenient online platform for participants (or caregivers) to self-report cases of HI in order to document the natural history and outcomes of individuals with HI.
- To improve knowledge of global prevalence of HI and any associated comorbidities.
- To better understand the role of timely diagnosis of HI on patient developmental outcomes.
- To better understand patient health outcomes of different HI treatment options, settings, and provider types.
- To identify both positive and negative effects related to different HI treatment options.
- To support the evolving standards of care for HI patients using natural history and outcome information from a global perspective.

The secondary objectives of HIGR are:

- To document the obstacles to accessing HI care, supplies, and medications.
- To measure the impact of HI and its management on patients’ and caregivers’ quality of life.
- To aid CHI and/or other country or region-specific HI patient organizations in identifying like genotypes or similar conditions to further connect HI patients/families within the larger HI community.
- To accelerate and facilitate HI clinical study development by identifying eligible research participants quickly and efficiently.
- To serve as an aggregated, de-identified resource to researchers seeking to study the pathophysiology of HI retrospectively in order to design prospective trials related to improving HI patient outcomes.
- To support the work of the CHI Collaborative Research Network (CRN) by providing natural history data and providing a platform for future research studies.

Individuals must consent to participate in the registry. The consent process can be completed by 1) an individual 18 years or older who is diagnosed with HI or suspected of having HI or 2) a parent or recognized legal guardian of a participant who is under the age of 18 or 18 years or older with assigned guardianship. The consented individual who completes the survey is referred to as the respondent. The study participant living with HI is referred to as the participant; in some cases, the respondent can also be the study participant. When a minor reaches the age of 18, that participant is asked to complete the adult consent process to authorize continuing access.

Investigators established a set of questions across surveys with predictable, congruent responses for any participant with an HI diagnosis. Investigators may identify participants whose responses are incongruent with the diagnosis and may need closer scrutiny. Potential outcomes of this type of review include outreach to the participant to clarify answers, exclusion from specific reports, or exclusion from the project.

Respondents provide consent for the use of de-identified data in research and communication preferences. Respondents can choose to be contacted by HIGR staff for networking opportunities and to learn about research studies. Since 2019, HIGR investigators have published an annual report to the community to share key highlights from the registry. These reports are a way to provide summaries of responses from critical surveys and allow participants to see how their responses match others in the community. Data is available to investigator-approved third-party researchers seeking to study the pathophysiology of HI retrospectively or to design prospective trials related to improving HI patient outcomes. Finally, de-identified information is shared with other large-scale health initiatives, such as the Rare Disease Cures Accelerator-Data and Analytics Platform, an FDA-funded initiative hosted by NORD and the Critical Path Institute (16). Individuals may withdraw their consent at any time.

The registry consists of thirteen surveys made up of questions about the patient’s experience with HI over their lifetime. An international team of HI experts, including family members of children with HI, advocates, clinicians, and researchers, developed the survey questions. The surveys are Contact Information, Demographics, Pregnancy, Birth, Diagnosis, Medication Management, Diet and Feeding Management, Surgical Management, Other Diagnoses, Glucose Monitoring Management, Development, Parent Quality of Life, and Patient Quality of Life. All surveys, except for Pregnancy and Birth, may be updated by the participant at their discretion when there is a notable change in their status, such as a new address, a change in treatment, or a newly diagnosed health condition. Respondents are asked to complete the longitudinal surveys, Glucose Monitoring Management and Quality of Life, every six months to provide information on the natural progression of the patient experience.

The registry team increases the accuracy and reliability of the registry data by sending respondents reminder emails to encourage them to update data and complete longitudinal surveys. HIGR investigators and staff monitor the data quality through automated and manual mechanisms. If the registry team determines one or more response(s) to be potentially invalid, an authorized staff member will contact the participant to clarify the response(s). The MaxHIGR sub-study, discussed below, will help confirm the overall validity and reliability of the data through the inclusion of physician generated data.

This paper shares trends observed through collected HIGR data from October 2018 to January 15, 2022. When answering surveys, individuals can answer as many or as few questions as they would like; therefore, the number of responses to questions may vary even within sections. If a question is updated or longitudinal, the most recent survey responses are reported, and descriptive statistics are provided.

## Results

### Participants

As of January 2022, 344 individuals consented to participate in HIGR, and 248 active respondents submitted a total of 2,192 surveys. Participants ranged from 2 weeks to 52 years old. Parents or guardians of individuals living with HI represented 83.9% of the individuals completing the surveys, 15.3% were individuals living with HI, and.8% identified as another category, including spouse and Aunt/Uncle. Individuals represented 46 different countries with 60.8% from North America, 23.8% from Europe, 6.7% from Asia, 4.1% from South America, 3.8% from Australia, and.9% from Africa.

### Subtype

Survey options for HI type include unknown, diffuse, focal, atypical, HI is suspected no formal diagnosis, and undiagnosed form of HI. A total of 171 individuals reported HI type. Diffuse HI is a general term that includes several forms of HI that affect the entire pancreas; 52.1% reported diffuse disease; 10.5% reported focal HI; 4.7% reported receiving an atypical HI diagnosis. Another 32.7% report one of the unspecified responses available, including HI suspected, unknown type, and undiagnosed form; the HIGR investigators further analyzed these individuals to confirm that they had HI based on positive genetic results or reported methods of medical management (**Table 1**).

**Table 1:** HI genetic testing results, by HI type.

	HI Genetic Testing Results		
HI Type	Positive for change/mutation in HI-related gene(s)n (%)	Negative for change/mutation in HI-related gene(s)n (%)	Totaln (%)
Diffuse	53 (61.6)	16 (37.2)	69 (53.5)
Focal	15 (17.4)	2 (4.7)	17 (13.2)
Atypical	5 (5.8)	1 (2.3)	6 (4.7)
Unknown	9 (10.5)	14 (32.6)	23 (17.8)
HI suspected	1 (1.2)	1 (2.3)	2 (1.6)
Undiagnosed form	3 (3.5)	9 (20.9)	12 (9.3)
**Total**	**86**	**43**	**129**

### Genetics and Syndromes

Of the 146 respondents who reported HI genetic testing, 69.2% of participants had positive results for a gene associated with HI, and 61.0% (n=89) had positive genetics on the first test. An additional 33 individuals had a second test and 36.4% (n=12) had positive results. Of the 129 respondents who provided HI type and genetic testing results, 66.7% of participants tested positive for a change/mutation in HI-related genes. The type of HI included 61.6% (n=53) diffuse disease. ABCC8 was the most commonly detected gene in 45.7% (n=59) of all participants who had genetic testing (**Table 2**).

**Table 2:** Detected HI genes.

Gene	Participants n (%)
ABCC8 (SUR1)	59 (62.1)
GLUD1 (Glutamate dehydrogenase)	13 (13.7)
KCNJ11 (KIR6.2)	9 (9.5)
GCK (Glucokinase)	5 (5.3)
HNF1A	3 (3.2)
SLC16A1 (MCT1)	2 (2.1)
HNF4A	1 (1.1)
HADH	1 (1.1)
UCP2	1 (1.1)
PMM2	1 (1.1)
**Total**	**95**

Individuals could choose more than one response.

Eleven participants had a HI-related syndrome. The syndromes include Beckwith-Weidemann, Kabuki, Turner, Sotos, Fanconi, Polycystic Kidney Disease, and Rubinstein-Taybi Type 2.

### Birth

Of newborns born with HI, 69.8% (n=90) were born term (37-42 weeks) with a mean weight of 3.75 kilogram (kg), 17.1% (n=22) were born between 34-36 weeks with a mean weight of 3.50 kg, and 12.4% (n=16) were born between 28-33 weeks with a mean weight of 2.46 kg (**Table 3**).

**Table 3:** Birth weight and gestational age.

Gestational Age	n (%)	Weight in KG	
		Mean (SD)​	[Min, Max]
Less than 28 weeks (extreme prematurity)	1 (.7)	1.25 (N/A)	N/A
28-33 weeks (prematurity)​	16 (12.4)	2.46 (.74)	[1.64, 3.88]
34-36 weeks (late prematurity)​	22 (17.1)	3.50 (.97)	[1.82, 5.19]
37-42 weeks (term)​	90 (69.8)	3.75 (.71)	[1.96, 5.64]
**Total participants**	**129**		

Of 170 newborns, 60% (n=102) had an abnormal blood glucose test recorded before leaving the birthing facility or being released into parental care, and 77.5% of them (n=79) reported abnormal blood glucose at one day old and 12.7% (n=13) reported abnormal blood glucose on day two. Of babies who had an abnormal blood glucose test, 50.0% (n=39) were in the normal weight range (**Table 4**). Of the newborns whose parents were aware of a blood glucose test, the age (in days) that the newborn first presented abnormal blood glucose was day one for 41.6% (n=79) of participants and day two for 6.8% (n=13).

**Table 4:** Abnormal blood glucose recorded before leaving facility, by birth weight.

	Birth Weight (kg)				
Parent reported results of an abnormal blood glucose test before leaving the birthing facility or before being released to parent care	Low birthweight (< 2.50)	Average birthweight (2.51 – 4.0)	High birthweight (4.01+)	Total	
Yes	8	39	31	78	
No	6	23	5	34	
Unknown	1	9	3	13	
Total	15	71	39	125	

Symptoms of hypoglycemia were first noticed for 22.0% (n=31) of all participants within the first hour of birth and an additional 18.4% (n=26) within 24 hours of birth (**Table 5**). Only 19.4% (n=33) of people reported having a fasting study before leaving the birthing facility.

**Table 5:** Age participant’s symptoms were first noticed.

Age participant's symptoms were first noticed	Participants n (%)
Unknown	3 (2.1)
Within 1 hour of birth	31 (22.0)
After 1 hour to 24 hours of birth	26 (18.4)
2 days of life	14 (10)
3 – 6 days of life	7 (5.0)
1 – 4 weeks of life	9 (6.4)
5 – 7 weeks of life	2 (1.4)
2 – 6 months old	27 (19.1)
7 – 12 months old	11 (7.8)
1 – 3 years old	7 (5)
4 – 9 years old	2 (1.4)
10 – 17 years old	1 (.7)
18 years +	1 (.7)
**Total**	**141**

No known risk factors of hypoglycemia were identified or reported shortly after delivery and before leaving the birthing facility for 28.4% (n=48) of newborns (**Table 6**). The risk factors included family history, hypoglycemia, and physical features. Only 4.7% reported a known family history of a genetic form of hypoglycemia.

**Table 6:** Identified signs of hypoglycemia before leaving the birthing facility.

Respondent reported known hypoglycemia risk factors identified prior to or shortly after delivery (before leaving the birthing facility)	Participants n (%)
Unknown	31 (18.3)
No risk factors	48 (28.4)
Family history of a genetic form of a hypoglycemia	8 (4.7)
Hemolytic disease of the newborn (break down of red blood cells)	0 (0)
Polycythemia (high concentration of red blood cells)	4 (2.4)
Abnormal physical features (e.g. midline facial malformations, microphallus)	2 (1.2)
Up to 48 hours of age: Unable to maintain sugar above 50 mg/dL (2,8 mmol/l or 0,5 g/L) before usual feed	80 (47.3)
Over 48 hours of age: Unable to maintain sugar above 60 mg/dL (3,3 mmol/l or 0,6 g/L) before usual feed	56 (33.1)
**Total**	**169**

Individuals could choose more than one response.

Before receiving a diagnosis of HI, 91.73% (n=132) of newborns were hospitalized for symptoms, including 33.9% (n=43) who were readmitted to the hospital following their initial stay at birth. The reported length of hospitalizations ranged from less than a week (9.5%) to 11-12 months (1.6%), with others reporting a hospital stay of 1-2 weeks (28.3%), 3-4 weeks (22.8%), 1-2 months (25.2%), or 3-4 months (13.4%).

Of the 127 newborns who had additional hospitalizations after the initial stay at birth before an HI diagnosis was made, 18.1% (n=23) were hospitalized one other time, 7.1% (n=9) were hospitalized two more times, and 8.7% (n=11) were hospitalized more than three times, including 2.4% (n=3) who were hospitalized more than ten times.

### Hospitalizations and Transition to Home

A total of 39.0% (n=55) of respondents reported a change in the participant’s medical treatment less than a month after discharge from the hospital after the initial diagnosis, and an additional 26.2% (n=37) reported a change in 1-3 months (**Table 7**). Examples of changes included medication type, dose or schedule, or a feeding regime or diet change. Only 8.5% (n=12) of respondents stated that no change was necessary.

**Table 7:** Changes in home management following HI diagnosis and pancreatectomy.

	Change in medical treatment after hospitalization where HI diagnosis was made n (%)	Change in HI management after pancreatectomy n (%)
No change necessary​	12 (8.5)​	7​ (22.6)
Less than 1 month​	55 (39.0)​	5​ (16.1)
1-3 months​	37 (26.2)​	11 (35.5)​
4-6 months​	19 (13.5)​	4​ (12.9)
7-9 months​	1 (.7)​	0 (0)
10-12 months​	2 (1.4)​	1 (3.2)
Greater than 1 year ​	7 (5.0)​	1 (3.2)
Unknown​	8 (5.7)​	2​ (6.5)
**Total​**	**141**​	**31​**

Within the past 12 months of when the respondents answered this question, 40.1% (n=68) of participants required hospitalization due to problems related to blood sugar; 20.6% required four or more stays, and 5.9% were hospitalized seven times or more.

### Medication Management

A total of 139 respondents completed the medication management survey and provided information about medication history. Participants have used the following HI medications: diazoxide (82.7%, n=115), octreotide (24.5%, n=34), glucagon (19.4%, n=27), and lanreotide (11.5%, n=16). Of participants whose medical management has included diazoxide, 65.2% (n=75) are currently taking the medication. Of those presently taking diazoxide, 12 respondents reported that participants had a low blood sugar below 70 mg/dL more than once a day, seven reported a low once per day, and five reported a low several times per week.

Of the participants who have ever used diazoxide, only 2.7% did not experience any side effects (**Table 8**). The most commonly reported side effects were increased growth of body hair (84.1%), loss of appetite (34.5%), swelling (hands, feet, or both) (25.7%), facial changes (23.9%), and stomach pain or upset (21.2%). A reported 29.2% of respondents for participants on diazoxide reported continued hypoglycemia. Of the 106 respondents who answered, 14.2% indicated that adverse effects caused the participant to stop taking the medication and 12.3% had to stop due to problems with availability.

**Table 8:** HI medication side effects.

Side effects	Diazoxide %	Octreotide %	Lanreotide %
None	2.7	30.3	25.0
Loss of appetite	34.5	N/A	N/A
Stomach pain or upset	21.2	24.2	18.8
Changes in sense of taste	8.0	N/A	N/A
Increase in growth of body hair	84.1	N/A	N/A
Headache	7.1	0	6.3
Dizziness	3.5	0	6.3
Skin rash	8.0	N/A	N/A
Facial changes	23.9	N/A	N/A
Swelling (hands, feet, or both)	25.7	N/A	N/A
Racing heartbeat (tachycardia)	15.0	N/A	N/A
Fluid in the lungs	5.3	N/A	N/A
Pulmonary hypertension	6.2	N/A	N/A
Continued hypoglycemia	29.2	42.4	43.8
Hyperglycemia	7.1	24.2	12.5
Nausea	.9	9.1	18.8
Changes in stool	N/A	33.3	43.8
Gallstone/gallbladder sludge	N/A	12.1	18.8
Growth suppression	N/A	6.1	0
Thyroid suppression	N/A	0	0
Necrotizing enterocolitis	N/A	0	0
Injection site problem	N/A	9.1	18.8
**Total **	**113**	**33**	**16**

Individuals could choose more than one response.

### Surgical Experience

Of 142 participants, 29.6% (n=42) had a pancreatectomy, and another 9.9% (n=14) of families considered a pancreatectomy, but the participant did not have surgery. Of these respondents whose child did not have surgery and reported a known HI type, all (n=11) had diffuse disease. For children who did not have a pancreatectomy, 76.9% of their parents said they preferred medical management, and 23.1% cited health concerns, such as diabetes or complications. Of participants who had a pancreatectomy, 21.4% (n=9) required an additional pancreatectomy.

The total amount of each participant’s pancreas removed was 95% or greater for 47.6% (n=20), between 50-94% for 26.2% (n=11), 25-49% for 11.9% (n=5), and less than 25% for the remaining 14.3% (n=6). Of the participants who had 95% or more of their pancreas removed, 25% (n=5) of respondents reported the use of pancreatic enzymes. Everyone over twelve years (n=8) old who had a subtotal pancreatectomy reported diabetes.

Of 31 participants, 16.1% (n=5) required a change in HI management less than one month after being released to go home after their first pancreatectomy (**Table 7**). An additional 35.5% (n=11) needed a change within 1-3 months. A change included a change in medication type, dose, or schedule, or feeding regime or diet. Only 22.6% (n=7) of respondents reported that no change in HI management was necessary.

### Glucose Monitoring

Continuous glucose monitoring systems (CGMs) were used by 45.7% (n=59) of participants, and 76.3% (n=45) of those individuals use CGMs continuously (**Table 9**). For those who responded that a Continuous Glucose Monitor (CGM) was not used, it was because they were not recommended by a health care professional (55.3%, n=42), the device was not covered under medical care benefits (18.4%, n=14), or the device was not affordable (13.2%, n=10).

**Table 9:** Frequency of glucometer and CGM use.

	How often does the participant wear the CGMs			
Average times per day participant uses a glucometer to monitor blood sugar	Continuously	Non-continuously	Does not use CGMs	Total
Less than once	5	4	12	21
1 time	7	1	10	18
2-3 times	11	4	29	44
4-6 times	12	3	15	30
7-10 times	8	1	4	13
More than 10 times	1	1	0	2
Unknown	1	0	0	1
**Total**	**45**	**14**	**70**	**129**

Of 131 participants, 98.5% did use a glucometer. The two individuals who did not use a glucometer used another device to monitor their blood sugar. A total of 53.5% of participants are either continuously using CGMs or are checking their blood sugar with a glucometer four or more times a day. The most common frequency of glucometer use was 2-3 times a day (34.1%, n=44); however, 11.6% (n=15) of participants check seven or more times a day. Additionally, 34.9% (n=45) of participants who are checking blood sugar with a glucometer continuously wear a CGM.

Twenty respondents reported blood sugars below 70 mg/dL (3.9 mmol/l, 0.7g/L) more than once a day. An additional 23 participants experience lows once per day or several times per week. In addition to lows, 14 participants experience high blood sugar above 180 mg/dL (10 mmol/L, 1.8 g/L) once per week or more frequently. This does not include participants with diabetes or focal HI.

As part of the participant’s HI management, 47.2% (n=67) of respondents said they have in-hospital fasting tests. 35.8% (n=24) said that the length of time the participant can fast in-hospital does not correspond to the length of time the participant can fast before blood sugar drops below 70 mg/dL (3.9 mmol/l, 0.7 g/L) at home, whereas 50.7% (n=34) of respondents believed it did correspond to their experience at home, and 13.4% (n=9) were unsure.

During episodes of blood sugar below 70 mg/dL (3.9 mmol/l, 0.7g/L), 29.3% of 123 respondents stated the participant never had symptoms, 33.3% said they rarely have symptoms, 24.5% sometimes have symptoms, 10.6% frequently have symptoms, and only 2.4% always have symptoms. Of the individuals that do experience symptoms of hypoglycemia, the most typical include irritability/mood changes (62.1%, n=54), lethargy/fatigue (51.7%, n=45), clouded thinking/confusion (32.2%, n=28), tremors/shakiness (25.3%, n=22), and headache (25.3%, n=22) (**Table 10**).

**Table 10:** Reported symptoms of hypoglycemia the participant exhibits when blood sugar is low.

What symptoms of hypoglycemia does the participant typically exhibit when blood sugar is low	Participants n (%)
Irritability/Mood Change	54 (62.1)
Anxiety	13 (14.9)
Loud crying	7 (8.0)
Lethargy/Fatigue	45 (51.7)
Headache	22 (25.3)
Clouded thinking/Confusion	28 (32.2)
Poor feeding/appetite	10 (11.5)
Constant hunger	14 (16.1)
Nausea	9 (10.3)
Unusual eye movement/Staring spells	18 (20.7)
Tremors/Shakiness	22 (25.3)
Sweating	21 (24.1)
Seizure	8 (9.2)
Other	9 (10.3)
**Total **	**87**

Individuals could choose more than one response.

### Diet and Feeding Management

Of 145 participants, 46.2% (n=67) used nasogastric or orogastric tubes and 32.4% (n=47) used a gastrostomy tube for feeds at some point since HI was diagnosed. Of the 81 participants who have utilized tube feeding to manage blood sugar, 50.6% (n=41) used them continuously. The majority, 69.4% (n=102) of participants, have between four to seven meals or snacks in an average 24-hour period by mouth or tube.

Of 137 respondents, 68.6% (n=94) report feeding issues, including 73.1% of participants with diffuse HI and 75.0% of participants with focal HI. Of the 30 participants with diffuse or focal HI who had a pancreatectomy, 80.0% had feeding issues (**Table 11**). Feeding issues include poor appetite (41.6%, n=57), refusing to eat (40.9%, n=56), reflux (32.1%, n=44), vomiting (27.7%, n=38), and problems with texture (28.0%, n=39).

**Table 11:** Feeding issues regularly experienced by participants.

Has the participant regularly experienced any feeding issues regularly	Participants n (%)
No feeding issues	43 (31)
Feeding issues	94 (67)
* Poor appetite*	57 (42)
* Refusing to eat*	56 (41)
* Reflux*	44 (32)
* Problems with texture*	39 (28)
* Gagging*	37 (27)
* Vomiting*	38 (28)
* Uncoordinated oral skills*	28 (20)
* Slow eating*	37 (27)
* Coughing*	23 (17)
* Overeating*	12 (9)
**Total**	**137**

Individuals could choose more than one response.

Of those who reported a feeding issue, 60.6% (n=57) said that the issue has not resolved. For the participants whose feeding issues have resolved, 33.3% (n=12) resolved within the first year of life, 30.6% (n=11) resolved between 1-3 years old, and 13.9% (n=5) were over the age of seven before the feeding issues resolved. For participants where the feeding issues have not resolved 36.8% (n=21) were between 0 to 1 year old, 36.8% (n=21) were between 2-5 years old, 15.8% (n=9) were between 6-15 years old, and 10.5% (n=6) are over the age of 16.

The reported average number of hours participants can fast without their blood sugar dropping below 70 mg/dL (3.9mmol/l, 0.7g/L) in the daytime and overnight varied considerably among the 123 individuals who responded (**Table 12**).

**Table 12:** Daytime and overnight fasting and low blood sugar.

Average number of hours participant can fast before his or her blood sugar drops below 70 mg/dL (3.9mmol/l, 0.7g/L)	Daytime n (%)	Overnight n (%)
0	4 (3.3)	3 (2.4)
1	4 (3.3)	0 (0)
2	9 (7.3)	4 (3.3)
3	22 (17.9)	7 (5.7)
4	30 (24.4)	7 (5.7)
5	10 (8.1)	4 (3.3)
6	7 (5.7)	8 (6.5)
7	0 (0)	4 (3.3)
8	4 (3.3)	10 (8.1)
9	1 (.8)	4 (3.3)
10	2 (1.6)	10 (8.1)
11	0 (0)	5 (4.1)
12	2 (1.6)	15 (12.2)
More than 12 hours	12 (9.8)	30 (24.4)
Unknown	16 (13.0)	12 (9.8)
**Total **	**123**	**123**

### Development and Neurology

Of 135 respondents who completed the developmental survey, 44.4% (n=60) reported delays in participant reaching developmental milestones. Of all respondents, 25.9% (n=35) reported a delay in talking/speech; other reported delays included walking (14.1%, n=19), feeding (13.3%, n=18), crawling (13.3%, n=18), and fine motor skills (11.9%, n=16) (**Table 13**).

**Table 13:** Reported developmental delays and neurological problems.

Reported neurological problems or delays in developmental milestones	Participants n (%)
Delays in reaching developmental milestones	135
* Talking/speech*	35 (25.9)
* Walking*	19 (14.1)
* Feeding*	18 (13.3)
* Crawling*	18 (13.3)
* Fine motor skills*	16 (11.9)
* Sitting*	12 (8.9)
* Motor skills*	3 (2.2)
* Gross motor skills*	9 (6.7)
* Rolling over*	6 (4.4)
* Standing*	4 (3.0)
* Globally delayed*	5 (3.7)
* Focus/attention*	2 (1.5)
* Adaptive skills*	5 (3.7)
* Reading*	2 (1.5)
Chronic neurologic problems felt to be due to prolonged hypoglycemia	130
* Developmental delay*	25 (19.2)
* Sensory processing*	25 (19.2)
* Learning disability*	13 (10.0)
* Poor coordination*	11 (8.5)
* Behavior problems*	10 (7.7)
* Seizure disorder*	9 (6.9)
* Hypotonia*	9 (6.9)
* Strabismus*	9 (6.9)
* Memory processing*	9 (6.9)
* Low vision*	5 (3.8)
* Other*	3 (2.3)
* Cerebral palsy*	3 (2.3)
* Movement disorder*	2 (1.5)
* Developmental delay*	25 (19.2)
Neurological disorders reported	141
* Epilepsy*	18 (12.8)
* Learning Disability*	14 (9.9)
* Anxiety*	12 (8.5)
* ADHD*	11 (7.8)
* Sensory Processing*	10 (7.1)
* Mental Health*	6 (4.3)
* Autism*	5 (3.5)
* OCD*	4 (2.8)
* Auditory Processing*	4 (2.8)
* Migraine*	3 (2.1)
* Cerebral Palsy*	3 (2.1)
* Depression*	3 (2.1)
* Dyslexia*	2 (1.4)
* Blindness*	1 (.7)
* Deafness*	1 (.7)
* Pervasive Disorder*	1 (.7)

For each question, individuals could choose more than one response.

Of 130 respondents, 37.7% (n=49) reported a chronic neurological problem they feel is due to prolonged hypoglycemia (**Table 13**). The problems include developmental delay (19.2%, n=25), sensory processing challenges (19.2%, n=25), learning disability (10.0%, n=13), poor coordination (8.5%, n=11), and behavior problems (7.7%, n=10).

A total of 31.2% (n=44) of respondents reported a neurological diagnosis, including 12.8% (n=18) of participants who have been diagnosed with epilepsy. Other reported diagnoses include learning disability (9.9%, n=14), anxiety (8.5%, n=12), ADHD (7.8%, n=11), and sensory processing (7.1%, n=10) (**Table 13**).

### Patient and Caregiver QOL

Of 23 adult participants, 52.2% (n=12) report they “very often” or “quite often” feel HI rules their lives, while 39.1% (n=9) report they “seldom” or “never” feel HI rules their lives. When asked to describe their experience with the daily management of the HI-related condition, adult participants and caregivers reported it was demanding, complicated, and disruptive (**Table 14**). Parents of children under 5 were more likely than parents of older children to indicate these responses. In contrast, a higher percentage of parents of older children indicated that the management of HI was simple (13% for parents of children under five versus 22.2% for parents of children over 12).

**Table 14:** Parent/caregiver and patient experience with daily management of HI.

	Parent/caregiver experience with daily management of the participant's HI	Participant experience with the daily management of their HI
Simple	22 (16.5)	5 (21.7)
Manageable	75 (56.4)	13 (56.5)
Demanding	56 (42.1)	7 (30.4)
Complicated	33 (24.8)	7 (30.4)
Disruptive	23 (17.3)	6 (26.1)
Other	2 (1.5)	0 (0)
**Total **	**133**	**23**

Individuals could choose more than one response.

The experience of obtaining a diagnosis was highly variable, with 27.6% (n=37) saying it was “very good,” but 22.4% (n=30) said it was “difficult,” and 18.7% (n=25) said it was “very difficult.”

The financial impact of HI had a varied effect on households; 31.6% (n=42) of respondents said their household income was negatively impacted “quite a lot” or “very much” due to the participant’s HI related condition, and only 11.3% (n=15) indicated they were not at all impacted. Of parents who responded, 62.1% (n=82) said that their work/career plans changed “somewhat,” “quite a lot,” or “very much” due to the participant’s HI-related condition. Additionally, 19.1% (n=25) of individuals said they missed work or school “quite a lot” or “very frequently” due to their child’s HI.

Of 133 parents, 31.6% (n=42) said they “always” worry because of the participant’s HI, and an additional 51.9% (n=69) said they worry “very” or “quite often” (**Table 15**). Despite these challenges, 19.4% (n=26) of respondents rated their quality of life as “excellent,” while 42.5% (n=57) said it was “very good,” 22.4% (n=30) said “good,” 13.4% (n=18) said “fair,” and only 2.2% (n=3) said it was “poor.”

**Table 15:** Parent/caregiver experience with worry because of the participants HI, by age groups.

Do you worry because of the participant's HI or HI-related condition	Under 5 years old	5 – 11 years old	12 years +	Total
Always	29	9	4	42
Very Often	11	12	6	29
Quite Often	19	9	12	40
Seldom	7	8	5	20
Never	2	0	0	2
**Total **	**68**	**38**	**27**	**133**

## Discussion

### Limitations

Most rare disease registries experience patient recruitment and retention challenges (5, 15), but CHI has made an effort to mitigate this through on-going engagement strategies, including presentations at family conferences, messages on social media, engagement with medical professionals to encourage recruitment, and direct email reminders. HIGR Participants are more likely to be from North America and younger than non-participants in the HI community. There is the potential for recall bias; however, rare disease patients and caregivers are very involved in disease management (17–19), which increases the likelihood of accurate responses.

Individuals who join the registry are likely to be more connected to CHI compared to those not participating, which could result in selection bias. Although there is some evidence generally in rare disease registries that participants have more severe forms of the diseases (13, 15), there is not enough information to evaluate if this is the case in HI. In the future we hope to recruit more individuals who have been cured, including transient patients and those who are post-pancreatectomy, as they are currently under-represented.

CHI is exploring other ways to collect data related to the overall burden of disease and experience of patients, combining these efforts with HIGR recruitment will provide a way to further characterize the burden of disease. Another limitation is that HIGR is currently only available in English. Therefore, the results may not be fully generalizable to all HI patients. However, plans are underway to include additional languages on the platform in the future.

In some cases, we report small numbers of cases, these are seen as individual vignettes of patient experiences that can act as a starting point to better understand the overall experience of living with HI. The HIGR team also has data validation and data quality standards to monitor the data’s reliability.

### Implications for HI Patients and Research

The early results of the insights generated by the only patient-reported data source are consistent with the literature and other studies completed, including the genetics (1, 2), the reported side-effects of current medications (3, 20, 21), the timing and development of post-pancreatectomy diabetes (22, 23), and the neurological implications associated with prolonged hypoglycemia (2–4).

The number of respondents who reported an unspecified type of HI, even when radiology, medical management, or surgery provided more information about type, indicates that the nomenclature of HI is not universally adopted in the patient community. Focal versus diffuse is important for surgery, especially since focal HI can be cured with surgery, but overall, these types may not resonate with patients because of the heterogeneity of severity in the diffuse group. It is important to understand what patients call their disease and to understand the overall experience across and within subtypes, research that can be furthered through the registry and natural history studies.

Of the newborns in HIGR, 30.2% were born premature. Annually, 11% of babies are born preterm globally (24). Therefore, the rate of preterm babies is much higher in HIGR than worldwide. According to the World Health Organization (WHO), the average weight of a full term baby is 3.2 kg for girls and 3.3 kg for boys, with a range of 2.5-4 kg (25). Babies that weigh less than 2.5 kg are considered low birth weight, regardless of gestational age. In the general population this is bottom 3^rd^ percentile of all babies, but in HIGR 12.4% of babies were under 2.5 kg. Babies over 4 kg are in the top 97^th^ percentile and 30.0% of all HI babies were born over 4 kg.

Additionally, this patient reported research adds many insights to the existing literature and provides new insights for timely diagnosis. Although babies born with HI were within the average weight ranges outlined by WHO, overall, they were on the higher end of the spectrum. In many hospitals, low weight and high weight babies may have their blood sugar screened, however, 50% of normal weight baby participants who were later diagnosed with HI did not have a blood glucose test before leaving the hospital. That normal weight babies in the general population are not typically screened at birth for low blood sugar increases the likelihood of neurologic sequelae and death in those born with HI. Furthermore, 33.9% of respondents reported hospitalization readmission for symptoms of hypoglycemia prior to diagnosis. Adopting the concept of blood sugar as a vital sign will help identify those newborns experiencing hypoglycemia. This is especially critical as 28.4% of newborns with HI in HIGR had no known risk factors of hypoglycemia before leaving the birthing facility.

HIGR also illuminates the on-going fluctuations in care during the transition from hospital to home and points to some of the reasons for these changes. It is revealing that only 19.4% of participants reported having a fasting study before leaving the birthing facility. Even for the individuals who had a fasting study, 66.7% required a change in management within three months. HIGR also shows the limits of in hospital fasting; 35.8% of individuals do not believe their in-hospital fasting experience was reflective of their experience at home.

After hospitalization, including for surgery, many respondents report a change in medical treatment within a few months of discharge, with 51.6% reporting a change within three months post-pancreatectomy and 65.2% reporting a change in medical treatment within three months of discharge from the hospital. This shows a need to prepare HI families for the on-going challenges of managing HI after they are discharged from the hospital and the need for constant vigilance by patients and families to monitor the patient’s condition.

The burden of finding a proper diagnosis and a successful disease management regime is reflected in the length of hospitalizations (three weeks or longer for over 62.2% of individuals) and the number of hospitalizations (three or more hospitalizations for 8.7% of all individuals). The burden of hospitalization is an on-going issue for individuals with HI, with 40.1% of respondents reporting at least one hospitalization within the past 12 months directly related to blood sugar.

One implication of the registry data is the need for new innovative treatments, as indicated by the reported side effects of current medications and the continued hypoglycemia among participants. One current categorization of HI in the literature is diazoxide responsive or diazoxide unresponsive; however, respondents report 32% of participants currently taking diazoxide experience low blood sugar below 70 mg/dL (3.9 mmol/l, 0.7g/L) multiple times a week or more frequently. This challenges the concept of diazoxide responsive and underscores the need for new options for medical management. Additionally, of 107 participants, 14.0% (n=15) had to stop taking diazoxide due to adverse effects.

Another insight is that for severe diffuse HI, a pancreatectomy is a choice fraught with significant negative health consequences. For those who have a subtotal pancreatectomy, individuals are trading one disease for another as eventually after their pancreatectomy, they develop diabetes and many also require pancreatic enzymes (22, 23, 26, 27). However, this tradeoff is often the only logical option to save the brain. These conditions may be an immediate change for some patients, while others may have eight years or more before diabetes occurs. This explains why individuals under twelve who underwent a subtotal pancreatectomy may not yet report diabetes.

HI patients are currently using glucose monitoring devices, such as CGMs by 43.4% of participants and glucometers by 98.5% of participants. Access to these devices is a problem, as is trust in CGMs by medical providers for some participants. Monitoring blood sugars is critically important, especially since only 13% of participants sometimes or frequently have symptoms when experiencing hypoglycemia. This underscores the overall reliance on blood sugar monitoring and the need to have accurate approved devices and tools to support patients and families.

Many individuals within the HI community have ongoing feeding issues, some requiring tube feeding regimes to stabilize the participant’s blood sugars either continuously (28.3% of all individuals) or overnight (26.9%). For 60% of participants, these feeding issues have not resolved. The feeding frequency is also an added part of the patient and family burden. In addition to those who required continuous feeds, many respondents report needing to feed every few hours to avoid drops in blood sugar, however, a constant challenge is refusal to eat or other feeding challenges. This represents a burden on the family as planning for meals needs to be a constant consideration.

The most-reported neurological outcomes of HI include 37.7% delays in developmental milestones and 31.2% receiving a specific neurological diagnosis. Swift action by medical professionals is key to reduce prolonged hypoglycemia, and increased awareness, and clinical guidance are crucial to prevent these and other issues.

On a positive note, parents describe a high quality of life despite these challenges. Parents are able to distinguish between burden of disease and happiness. Parental descriptions of ongoing worry, the demands of the disease, and financial impacts provide more insight into the burden of disease that accompanies the overall fears of hypoglycemia and the burden balancing complex medical and feeding regimes. For those whose children were diagnosed within the first few days of life, living with a child diagnosed with a rare disease is all the child and their family know, and rare disease families are known for their resilience.

### Future Directions

In addition to the potential of the CRN to provide infrastructure to facilitate partnerships with researchers at different institutions and companies to explore research questions that can be explored with the registry in the future, some specific projects are already underway. Any academic researcher can request data from HIGR to support their research.

Two pilot projects are supported through the Million Dollar Bike Ride (MDBR), a project of the Orphan Disease Center at the University of Pennsylvania. The 2020 pilot grant is funding MaxHIGR, a partnership between researchers at Northern Congenital Hyperinsulinism Service (Manchester, England), Collaborative Alliance on Congenital Hyperinsulinism (Magdeburg, Germany), Great Ormond Street Hospital for Children (London, England), The Children’s Hospital of Philadelphia (Pennsylvania, USA), Cook Children’s Hospital (Texas, USA), University Medical Center - National Research Center for Maternal and Child Health (Nur-Sultan, Kazakhstan), other academic researchers worldwide, and CHI to include physician-reported data elements in HIGR. The project is being conducted as a pilot to expand the data generated through HIGR and increase the universe of information available to researchers and regulatory bodies in the future. MaxHIGR will allow for the possibility of validating the patient-reported dataset, thus enhancing the value, credibility, and impact of HIGR. The secondary aims of MaxHIGR are to validate the parent QOL questionnaires clinically and build a prototype model for an integrated database, combining fields from patient-populated and clinician-provided information.

The 2021 MDBR recipients from the Children’s Hospital of Philadelphia are also utilizing HIGR as part of a multi-center observational study to investigate the natural history of hyperinsulinism hyperammonemia syndrome. The goal is to increase understanding of disease and outcomes for patients.

To further support needed HI research, the HIGR investigators are expanding the scope of data collection to include CGM data. Blood sugar levels are of critical importance to HI patients. Understanding patterns alongside other patient-reported outcomes from a wide range of patients is valuable to clinicians, drug developers, and families. Many patients use CGMs to monitor their blood sugar and rely on the insights provided to manage their disease and prevent lows. Currently, CGMs are not approved for use in HI. Generating additional evidence related to their use and impact on disease management could help lead to device designation and increased access for HI families.

A final additional avenue for HIGR is to aid in the drug development process more directly by collecting post-marketing information and as a control arm in clinical trials. The registry can create substudies to follow a subset of patients, such as individuals on a specific treatment, and to collect existing or specifically developed surveys. Looking at sub-populations and their experience across time can provide a naturally occurring control group for a clinical trial. Controls are essential for scientific understanding, but there are ethical implications for not allowing individuals to try potential new treatments in a rare disease clinical research study. In HI, it is very difficult to conduct blinded studies as without the standard of care therapy individuals would likely experience hypoglycemia from the very beginning of the study and throughout the duration. Additionally, as previously discussed, a participant’s medical management and needs change frequently, which makes it difficult to even use prior historical data from the individuals’ own experience as a control. A larger pool of historical data or active controls from individuals who cannot logistically join an active study can help provide the needed evidence. The FDA has provided draft guidance to industry to utilize registries in this way (11), but patient organization registries are uniquely positioned to support trials and should receive similar guidance and support to further clinical development within their disease state.

## Conclusion

HIGR has already shown the potential to capture and disseminate information that can generate new insights into HI, including patient outcomes and caregiver QOL. The data presented above underscores the need for new treatments for individuals that have fewer side effects and reduce hypoglycemia events. CHI also advocates for the adoption of blood sugar as a vital sign, to further identify and diagnose patients and prevent brain damage. This information can help drive new research for treatments and cures and support clinical trials. Understanding the natural history of the disease can also guide standards of care. The data generated through HIGR provides an opportunity to improve the lives of all those affected by HI.

## Data Availability Statement

The raw data supporting the conclusions of this article will be made available by the authors, without undue reservation.

## Ethics Statement

The studies involving human participants were reviewed and approved by North Star Review Board. The patients/participants provided their written informed consent to participate in this study.

## Author Contributions

TP determined which data elements to include in the manuscript, directed the data analysis process, and wrote the initial draft. She was involved in all revisions to the text. JR, together with the other founding investigators, conceptualized and designed the questionnaires in HIGR. JR was the senior author on this paper leading the interpretation and contextualization of key findings in the paper and conducted major revisions and edits to the text. MM generated data reports, analysis, and tables for the manuscript and provided text for the future directions section of the paper. JS provided comments and critical revisions of the manuscript. All authors read and approved the final manuscript. All authors contributed to the article and approved the submitted version.

## Funding

Crinetics Pharmaceuticals, Eiger Biopharmaceuticals, Hanmi Pharmaceuticals, Rezolute, and Zealand Pharma are current financial sponsors of the HI Global Registry. The CHI Collaborative Research Network is funded through the Chan Zuckerberg Initiative Rare as One Network grant program (grant number: 2019-2111572). CHI has many individual and foundation donors funding other CHI projects and infrastructure.

## Conflict of Interest

All authors are employees of Congenital Hyperinsulinism International (CHI). CHI receives sponsorships for events from Amidebio, Betabionics, Crinetics Pharmaceuticals, Eiger Biopharmaceuticals, Hanmi, Rezolute, Twist and Zealand Pharma over the past 3 years. Crinetics Pharmaceuticals, Eiger Biopharmaceuticals, Hanmi Pharmaceuticals, Rezolute and Zealand Pharma are current sponsors of the HI Global Registry. None of the authors receive any fees directly from the companies. CHI staff and the board of directors retain all control of the programmatic aspects of these programs.

## Publisher’s Note

All claims expressed in this article are solely those of the authors and do not necessarily represent those of their affiliated organizations, or those of the publisher, the editors and the reviewers. Any product that may be evaluated in this article, or claim that may be made by its manufacturer, is not guaranteed or endorsed by the publisher.
